# Abuse liability for esketamine in a cohort of patients undergoing an acute treatment course to manage treatment-resistant depression: a secondary analysis of an observational study in real-world clinical practicee

**DOI:** 10.1177/20420986251347360

**Published:** 2025-06-19

**Authors:** Gilmar Gutierrez, Gustavo Vazquez, Nisha Ravindran, Raymond W. Lam, Peter Giacobbe, Karthikeyan Ganapathy, Annette Kowara, André Do, Anusha Baskaran, Sean Michael Nestor, Jennifer Swainson

**Affiliations:** Department of Psychiatry, University of Alberta, Edmonton, AB, Canada; Department of Psychiatry, Providence Care, Queen’s University, Kingston, ON, Canada; Centre for Neuroscience Studies, Queen’s University, Kingston, ON, Canada; Providence Care Hospital, Kingston, ON, Canada; Department of Psychiatry, University of Toronto, Toronto, ON, Canada; Centre for Addiction and Mental Health, Toronto, ON, Canada; Department of Psychiatry, University of British Columbia, Vancouver, BC, Canada; Department of Psychiatry, University of Toronto, Toronto, ON, Canada; Sunnybrook Health Sciences Centre, Toronto, ON, Canada; Alberta Hospital Edmonton/Envision Mind Care, Edmonton, AB, Canada; Department of Psychiatry, University of Toronto, Toronto, ON, Canada; Centre for Addiction and Mental Health, Toronto, ON, Canada; Department of Psychiatry, University of British Columbia, Vancouver, BC, Canada; Department of Psychiatry, Université de Montréal, Montreal, QC, Canada; Department of Psychiatry, University of Toronto, Toronto, ON, Canada; Sunnybrook Health Sciences Centre, Toronto, ON, Canada; Department of Psychiatry, University of Toronto, Toronto, ON, Canada; Sunnybrook Health Sciences Centre, Toronto, ON, Canada; Jennifer Swainson Department of Psychiatry, University of Alberta; Neuroscience and Mental Health Institute, 3rd floor, Cabrini Center, c/o Misericordia Community Hospital, 16940 87 Avenue NW, Edmonton, AB T5R 4H5, Canada

**Keywords:** abuse liability, antidepressant effect, ketamine abuse, major depressive disorder, psychopharmacology, real-world clinical data, suicidal ideation, treatment response

## Abstract

**Background:**

Intranasal (IN) esketamine has become an effective and well-tolerated therapeutic option for the management of treatment-resistant depression within major depressive disorder (MDD-TRD). Despite these promising benefits, given the prevalence of ketamine abuse, concerns remain over the addiction liability that may be associated with esketamine treatment.

**Objectives:**

The objective of this study is to assess the real-world abuse liability of this substance by tracking changes in likeability and cravings through an acute treatment course.

**Design:**

This is a secondary analysis of a previously published multicenter observational study.

**Methods:**

Likeability and craving for esketamine were assessed using the Likeability and Cravings Questionnaire (LCQ) in MDD-TRD patients receiving an acute course of IN esketamine treatment (eight dosing sessions). The data were analyzed using descriptive statistics, multivariate analysis of variance (MANOVA), and pre-post effect size (Cohen’s *d*).

**Results:**

Twenty-three patients (52.2% female, 43.5 ± 11.9 years old) were assessed. Most patients reported a neutral liking and no cravings for esketamine after their first dosing session. These metrics did not increase significantly by treatment endpoint. MANOVA showed that neither age, sex, baseline depression scores, the presence of side effects, or the study site had a statistically significant impact on LCQ scores either alone or in combination.

**Conclusion:**

These results agree with the available literature, showing that an acute course of IN esketamine treatment was not associated with high levels of drug liking or cravings, and this did not increase through the course of eight treatments. Though larger studies are needed, esketamine does not appear to be associated with significant abuse liability when used in an acute course of treatment for patients with MDD-TRD. These are important results for this patient population and for clinical practice.

## Plain language summary

An observational study assessing changes in esketamine liking and cravings through a 4-week treatment course of patients with treatment resistant depression

Depression is a serious mental illness that can be difficult to treat in some patients. Intranasal esketamine is a newer agent that can be added to an antidepressant to enhance the response. Esketamine is a drug derived from ketamine, which is a medication that has been used for many years in anesthesia and pain medicine, It is also a drug that can be abused or misused. One concern about medical use of esketamine is whether patients would “like” its psychological effects, or begin to crave it. This would be concerning as it may lead to misuse, over use, or seeking black market ketamine. To our knowledge, this is the first study that evaluates level of drug liking and craving along with improvement in mood in real world patients treated with esketamine. Twenty nine patients completed drug liking/craving and mood questionnaires through their course of esketamine treatments. Results are reassuring because most patients rated a neutral “liking” and minimal or no “craving” for esketamine, even after repeated treatments. While clinicians still must be cautious in use of esketamine, this study suggests that the medication is not likely to be abused or misused by patients taking it for depression.

## Introduction

Given the disease burden, prevalence, and loss of quality of life associated with major depressive disorder (MDD),^1,2^ and the sometimes-unsatisfactory response of current antidepressant treatment options,^3,4^ novel treatments such as ketamine and esketamine have emerged as promising alternatives for the management of major depressive disorder with treatment-resistant depression (MDD-TRD).^5–7^ An estimated 1/3 of patients fail to respond to conventional antidepressant treatments,^3,4^ supporting the approval of intranasal (IN) esketamine for the treatment of depression that fails to respond to two or more antidepressants in Canada, the United States, and many European countries. In addition, though off-label, both IV and non-IV forms of ketamine have been recognized by treatment guidelines as effective treatment options.^3,4,8–10^ Furthermore, ketamine and esketamine treatments have shown potential effectiveness in diverse psychiatric conditions, including bipolar disorder (impacting around 50% of TRD cases initially diagnosed as MDD-TRD) and substance use disorder (SUD).^11–16^ Despite the recognition and growing interest in the implementation of esketamine and ketamine-based treatments,^3,4,9^ concerns remain over the potential harms, such as the abuse liability of these substances.^17–19^

A previous scoping review^19^ that included 55 preclinical studies reported that although (*R*)-ketamine did not appear to carry abuse liability in rodents, animal data suggested abuse potential of both (*S*)-ketamine and racemic (*R, S*)-ketamine. While (*S*)-ketamine (esketamine) and racemic ketamine are the current forms available to treat depression, the reviews of clinical data have not reported significant concerns with misuse, dependence, or diversion when used in populations with MDD-TRD.^18,19^ The absence of reported concern, however, is primarily reliant on an absence of patient/participant reports, and few studies aimed to actively evaluate the abuse liability of these treatments.^17–19^

Though esketamine is tightly regulated through a risk evaluation mitigation system, to date, no studies have actively evaluated its true abuse liability. Studies for racemic ketamine have presented reassuring results, reporting a low risk of developing cravings for these substances when used therapeutically.^17–20^ For instance, a survey conducted by Feifel et al.,^21^ reported that adult patients receiving ketamine treatment for MDD-TRD did not report the development of addiction symptoms associated with this treatment, but this relied on a survey of ketamine providers. In a study by Chubbs et al.,^17^ actively surveyed patients with MDD-TRD taking maintenance IN or sublingual ketamine, to evaluate levels of drug “liking” and “craving.” Few patients reported high drug liking, and several patients actually disliked the effects of ketamine but continued to use it for its antidepressant effects. A few individuals endorsed wanting to use more than prescribed but clarified this was not for pleasurable psychoactive experiences, but because they perceived that more ketamine may further help their mood disorder.^17^ Importantly, in the context of addictions, the Food and Drug Administration, has highlighted that subjective drug “liking” and cravings are critical markers of addiction risk.^17^

Nevertheless, outside of a clinical or therapeutic setting, the abuse liability and consequences of ketamine abuse are well documented in the literature. Ketamine abuse was first reported in the late 1960s, with its use becoming common in nightclubs by the mid-1990s. Ketamine misuse and the resulting visual hallucinations and dissociation have been reported as the main factors promoting ketamine addiction and abuse.^17–19^ Though cumulative exposure of ketamine in drug abusers is far greater than that when used to treat depression,^20^ long-term ketamine abuse has been linked with several health consequences, including urological toxicity and interstitial cystitis, hydronephrosis, abdominal pain, liver injury, and cognitive impairments.

From a neurobiological perspective, Swainson et al.^18^ reviewed mechanisms suggested in the literature to influence ketamine’s addictive potential. The multiple pathways that have been raised as relevant include opioid system activation, stress hormone pathway dysregulation (including adrenocorticotropic hormone and cortisol-level increase), oxytocin reduction (also seen in other SUD), orexin-A reduction (linked to addictive potential), limbic system activation, and cortical atrophy causing impaired inhibition of addictive behaviors.^18,22^ An older report draws connections between chronic ketamine abuse and elevated levels of serum brain-derived neurotrophic factor (BDNF), which subsequently are involved in the modulation of midbrain dopamine reward circuits. Though the weekly amount of ketamine in this report was not clearly quantified, the authors also noted the role of BDNF/dopamine interaction in ketamine’s antidepressant effects. Interestingly, elevated BDNF levels have also been found in methamphetamine and ecstasy abusers, so it would seem that this BDNF/dopamine interaction may be complex and play a role in both antidepressant effects and the brain’s dopamine reward pathways, the latter of which may conceivably elevate or reduce substance abuse risks.^23^ Interestingly, the literature has shown that chronic antidepressant use also results in elevated serum BDNF, while a low serum BDNF has been associated with a variety of psychiatric conditions, including depression, anxiety, and SUD.^23,24^

Further to the preclinical literature suggesting a lower abuse liability for (*R*)-ketamine than (*S*)-ketamine or racemic ketamine, a study by Bonaventura et al.,^25^ found that (*S*)-ketamine and the (*R*)-ketamine (arketamine) have different affinities for brain receptors that can mediate addictive liability. Esketamine has a higher affinity for the N-methyl-D-aspartate (NMDA) receptor and the opioid receptors, while arketamine has a higher affinity for the sigma-1 and sigma-2 receptors. Authors suggested that this difference in affinity may indicate that esketamine has a higher abuse liability than arketamine, especially in patients with comorbid SUD.^25^

The serious consequences of ketamine abuse have resulted in strict federally regulated guidelines for access to IN esketamine treatments both in Canada and the United States.^17–19^ The available data have shown that ketamine has a similar addiction liability as esketamine.^17–20^ While Janssen’s clinical trials for IN esketamine did not assess drug liking or cravings in their study participants with MDD-TRD, the effects of esketamine were “liked” by polysubstance users.^26^ These results, and the scarcity of studies on the abuse liability of esketamine and ketamine-based treatments, highlight the need for additional studies assessing this risk, to help support the safe implementation of these treatments.

To address this research gap, a secondary analysis of a previously published multicenter study^27^ was conducted to further elucidate the abuse liability of IN esketamine treatment in patients undergoing an acute treatment course to manage TRD. Given that previous literature did not find evidence of significant abuse liability of ketamine or esketamine in the MDD-TRD population,^17–20^ this study hypothesized that patients receiving IN esketamine treatment will not have significant drug liking or cravings and that these will not increase by treatment endpoint.

## Methods

### Setting and participants

This is a secondary analysis of a previously published multicenter prospective observational cohort study^27^ of patients who received an acute course of IN esketamine treatment at Envision Mind Care in Edmonton, AB; Providence Care Hospital in Kingston, ON; Centre for Addiction and Mental Health in Toronto, ON; the Djavad Mowafaghian Centre for Brain Health in Vancouver, BC; and Sunnybrook Health Sciences Centre in Toronto, ON. Participants included adult (18–65 years old) outpatients experiencing major depressive episodes as determined by board-certified psychiatrists using DSM5-TR criteria and characterized as MDD-TRD (i.e., failed at least two antidepressant treatments of adequate dose and duration^3,4,9^). In addition, following standard-of-care practices, patients with psychosis, a primary diagnosis of personality disorder, uncontrolled hypertension, active substance abuse (i.e., in the past 6 months), current pregnancy or breastfeeding status, or those who had a previous negative reaction to ketamine, were ineligible to receive IN esketamine and participate in this study.^3,9^ Importantly, since this is an observational study of naturalistic clinical practice, it is worth clarifying that this study did not recruit participants to receive IN esketamine treatment and that the research team was not involved in the assessment of IN esketamine treatment eligibility, instead this study focused exclusively on the collection of observational data from patients already deemed eligible to receive IN esketamine treatment by their most responsible physician.^28,29^

IN esketamine was provided following well-established criteria supported by the Canadian Network for Mood and Anxiety Treatments (CANMAT) recommendations^3,9^ as an add-on treatment to the patient’s current psychopharmacology regimen. These criteria included discontinuing medications with the potential to interact with esketamine prior to the start of the treatment and for the duration of treatment. This process was monitored by the most responsible physician for each patient.^3,30^ These medications included benzodiazepines, naltrexone, lamotrigine, gabapentin, and pregabalin.^3,28,31,32^

The Health Sciences Research Ethics Board at Queen’s University (TRAQ #: 6031788) and institutional review boards at the collaborating sites reviewed and approved this research study. Participants in this study provided voluntary, written informed consent for the collection, analysis, and presentation of aggregate data. This study adhered to the standards set by the Declaration of Helsinki, and data management complies with US Federal Health Insurance Portability and Accountability Act (HIPAA) regulations pertaining to patient record confidentiality. The reporting of this study conforms to the STROBE statement.^33^

### IN esketamine treatment

Following standard-of-care clinical guidelines, eligible patients received an acute course of IN esketamine consisting of twice-a-week dosing sessions for 4 weeks.^3,4^ The treatments were administered and supervised by a healthcare professional. Clinic staff recorded any side effects present during and after IN esketamine administration at regular intervals, including changes in blood pressure, oxygen levels, onset, duration, and severity of common side effects.^28,30,34^ Esketamine was administered as a nasal spray, two sprays per nostril (56 mg total) for the first treatment and three sprays per nostril (84 mg) for the rest of the acute series. For the dosing session, patients were asked to fast for a minimum 2 h prior to esketamine administration and were instructed to stay resting in the treatment room until 30 min post-administration. Patients were discharged once they returned to a calm, oriented, and alert state and provided that their vitals were back to baseline and any side effects had dissipated.

### Data collection and outcome measures

This study presents a secondary analysis of the sample reported in a previous study,^27^ considering only the patients who completed their baseline Likeability and Craving Questionnaire (LCQ). As this was a naturalistic study collecting data from routine clinical practice, six patients from the original sample did not complete a baseline LCQ, so they were excluded from this analysis. Two additional patients who completed their IN esketamine treatment after the publication of the previous study were included in this report. The previous study did not consider the analysis of LCQ since it was not available for all patients, and the focus was on the real-world efficacy and tolerability of IN esketamine for MDD-TRD.

The aims of this part of the study involved determining whether repeated therapeutic IN esketamine administrations resulted in patients craving or liking esketamine for purposes beyond the management of their depression symptoms. This was assessed using the LCQ developed by Wang et al.,^26^ which has three questions scored on a scale from 0 (lowest) to 10 (highest)—one question for likeability of esketamine’s psychoactive effects (LCQ1), and two for esketamine craving assessing misuse (LCQ2) and abuse (LCQ3) liability respectively. Two measurements were taken per acute treatment course, baseline LCQ was assessed up to 24 h after the first IN esketamine dosing session, and the second assessment was completed up to 24 h after the last IN esketamine dosing session (eighth session—treatment endpoint). Our analysis then focused on determining whether there was a statistically significant change in any of the three LCQ questions when comparing the baseline and treatment endpoint scores. The LCQ assessments were conducted over the phone or in-person.

While not a validated scale, the LCQ was chosen as the only available questionnaire specifically designed to address the abuse liability of ketamine or esketamine, and it encompasses risk factors identified as important by the United States Food and Drug Administration.^26^ The LCQ had previously been used to assess the abuse liability of racemic ketamine in patients with MDD-TRD,^17^ thus use of the same questionnaire could allow for comparisons of ketamine and esketamine in clinical populations.

### Data analysis

The statistical analysis of the collected data considered both intention-to-treat (ITT) and per-protocol (only treatment completers) analytic approaches. Multiple imputation was applied to address missing data points, either due to incomplete or missing assessments or patient dropout.^35^ The pattern of missing data was random, and a Mersenne Twister algorithm was applied to generate random numbers. Following this multiple imputation approach, the model conducted five multiple imputation iterations using linear regression as the imputation method. The pooled result from the five multiple imputation iterations was used in the calculation.^36^

The LCQ data were analyzed using descriptive statistics for both ITT and per-protocol data to compare score changes between the start of the IN esketamine treatment and the treatment endpoint (week 8).^37–40^ Effect size was calculated using Cohen’s *d* with a 95% confidence interval (95% CI).^36,41^ A histogram with error bars was used to represent the primary outcome measures for each LCQ question at the two assessment time points.^37–40^

The impact of age, sex, baseline depression scores, antidepressant treatment response, presence of side effects, and study site on LCQ scores were analyzed using one-way and two-way multivariate analysis of variance (MANOVA). This assessment considered the descriptive statistical analysis conducted on the data, the use of multiple imputations to address missing data, assumed multivariate normality, homogeneity of covariance matrices, and linearity (pre-analysis); and employed Wilk’s Lambda to test for between-group differences in the multivariate analysis. Post hoc analysis employed Tukey’s Honest Significant Difference.^36,41^ All analyses were conducted on IBM SPSS Statistics for Mac, version 24 (IBM Corp., Armonk, NY, USA) at a significance level of *p* < 0.05.^36^

## Results

Twenty-three participants receiving IN esketamine treatment for the management of MDD-TRD from March 2023 to January 2024 were recruited for this study, seven from Envision Mind Care, five from Providence Care Hospital, eight from Sunnybrook Health Sciences Centre, and one from the Centre for Addiction and Mental Health. Data from two additional patients treated at Envision Mind Care subsequent to the analysis of the original study’s data were included in this analysis. Two participants dropped out of esketamine treatment (8.7%), and thus dropped out of the study. None of the patients mentioned symptom exacerbation or side effect burden as a reason for dropping out. Instead, the reasons for dropping out included challenges attending regular in-person session appointments, lack of symptom improvement, and personal preferences. Participant sociodemographic information is presented in Table 1.

**Table 1.** Participant sociodemographic characteristics and side effect profile.

Variable	IN esketamine (*n* = 23)
	Mean (SD) or %
Age	43.5 (11.9)
Sex (%Female:Male)	52.2:47.8%
Baseline depression	33.0 (7.3)*
Baseline LCQ	
LCQ1	4.6 (2.5)
LCQ2	0.6 (1.5)
LCQ3	0.2 (0.7)

* Depression score recorded with the Montgomery-Åsberg Depression Rating Scale.

LCQ, Likeability and Craving Questionnaire.

### Changes in likeability and craving for IN esketamine treatment

**Figure 1.**
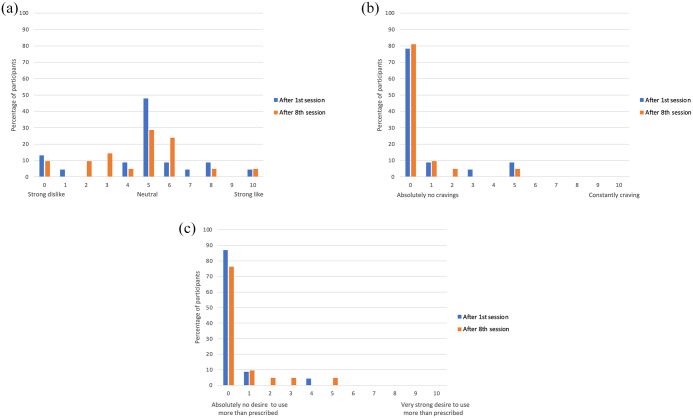
Frequency of LCQ scores after the first and last IN esketamine dosing session for LCQ1 (a), LCQ2 (b), and LCQ3 (c). LCQ, Likeability and Craving Questionnaire.

Analysis of LCQ data showed that liking and craving for esketamine did not change significantly from the first session to the treatment endpoint (eighth IN esketamine dosing session). The majority of patients reported no changes or a decrease in liking and craving for esketamine by treatment endpoint. MANOVA showed that neither age, sex, baseline depression scores nor study site had a statistically significant impact on LCQ scores either alone or in combination (Wilk’s Lambda and between-subject effects for age, sex, baseline depression scores, presence of side effects, and study site were nonsignificant). Table 2 presents the descriptive statistical analysis for the primary and secondary outcomes. Similar results were found in the per-protocol and ITT analyses. Figure 1 presents a histogram showing the frequency of LCQ scores at baseline (after the first IN esketamine session) and treatment endpoint.

**Table 2.** Descriptive statistical analysis of LCQ scale following a per-protocol and intention-to-treat analysis.

Scales	*n*	Pre (*m*1)	SD	Post (*m*2)	SD	*m*2 − *m*1	Effect size	*p*	Effect size (95% CI)
Per-protocol analysis									
LCQ1	21	4.6	2.5	4.5	2.4	−0.2	0.1	0.8	−0.5; 0.6
LCQ2	21	0.6	1.5	0.4	1.2	−0.2	0.1	0.6	−0.4; 0.7
LCQ3	21	0.2	0.7	0.6	1.3	0.3	0.3	0.3	−0.3; 0.9
Intention-to-treat analysis									
LCQ1	23	4.6	2.5	4.4	2.4	−0.2	0.1	0.7	−0.5; 0.7
LCQ2	23	0.7	1.5	0.4	1.1	−0.2	0.1	0.6	−0.4; 0.7
LCQ3	23	0.2	0.7	0.6	1.2	0.4	0.4	0.2	−0.2; 0.9
Percentage of patients experiencing LCQ score changes between 1st and last IN esketamine dosing session									
		LCQ1 (%)			LCQ2 (%)			LCQ3 (%)	
Increase		33.3			9.5			14.3	
No change		28.6			71.4			76.2	
Decrease		38.1			19.1			9.5	

LCQ, Likeability and Craving Questionnaire.

1. Assessment of esketamine likeability (LCQ1)

Most participants reported a neutral liking (score of 5: LCQ1 = 47.8%) for esketamine after the first dosing session. These score distributions did not change significantly at the treatment endpoint. The largest score change reported by patients who experienced an increase in LCQ metrics by treatment endpoint were four points for LCQ1 reported by one patient. This patient had a score of 0 in LCQ1 after the first IN esketamine dosing session, which suggests negative drug liking or dislike which moved to a more neutral experience at the end of treatment. Only one patient reported a “strong liking” for esketamine, and this remained unchanged by the treatment endpoint. No other patients developed a strong liking.

2. Assessment of esketamine cravings (LCQ2)

Most participants reported no cravings (score of 0: LCQ2 = 78.3%). These score distributions did not change significantly at the treatment endpoint. The largest score change reported by patients who experienced an increase in LCQ metrics by treatment endpoint were one point for LCQ2 reported by two patients. These patients had a score of 0 in LCQ2 after the first IN esketamine dosing session.

3. Assessment of desire to use esketamine in higher amounts than prescribed (LCQ3)

Most participants reported no desire to use more esketamine than prescribed (score of 0: LCQ3 = 86.96%). These score distributions did not change significantly at the treatment endpoint. The largest score change reported by patients who experienced an increase in LCQ metrics by treatment endpoint were five points for LCQ3 reported by one patient. This patient had a score of 0 in LCQ3 after the first IN esketamine dosing session.

## Discussion

The results of this study suggest that when used therapeutically, IN esketamine treatment was not associated with significant drug liking, craving, or desire to use more than prescribed, nor did it significantly increase with repeated administration of the drug over eight sessions.^31,43–51^ The analysis also suggested that neither the changes in likeability nor cravings were impacted by the age, sex, or baseline depression scores of the patients, or the study site.

Currently, studies assessing the abuse liability of IN esketamine treatment are scarce,^17–19^ which highlight the importance of this research study to further elucidate the safety of this treatment. For instance, in agreement with the results of this study, a study by Wajs et al.^52^ looking into the effectiveness of IN esketamine treatment in addition to an oral antidepressant for the treatment of patients with MDD-TRD, reported that none of their participants sought to use, abused, or requested to increase the frequency or dose of esketamine throughout the treatment course (4 weeks acute course and 1-year maintenance treatment). Importantly, however, the Wajs et al.^52^ study only tracked these metrics through the spontaneous subjective reports of their participants, instead of actively questioning key markers such as likeability or cravings using a consistent scale or questionnaire for all participants. Though clinical trials have not demonstrated significant risk, one case report by Orsolini et al.,^53^ described a 34-year-old woman with MDD-TRD who developed drug-seeking behaviors and craving symptomatology after receiving esketamine treatment. Slow esketamine de-titration and the addition of bupropion led to favorable results in the management of the addiction symptoms of this patient.^53^

A previous report by Chubbs et al.^17^ with MDD-TRD patients receiving sublingual or IN ketamine for an average of 41.2 weeks, found that “liking” of the ketamine experience was not universal. Using the LCQ, this report found that of 33 patients, 9 reported a neutral liking and 7 reported negative drug liking. Of the 17 respondents who had a positive liking for ketamine, cravings were present to some degree in about half. These results agree with our findings, which showed that nearly half (47.8%) of participants had a neutral liking for esketamine, and a few participants reported an increase in liking and cravings for esketamine by treatment endpoint.

One participant in our study went from a score of 0 (no desire) after the first esketamine treatment, to a score of 5 (moderate desire) at the treatment endpoint, in terms of desire to use esketamine in greater amounts than prescribed (LCQ3). Likewise, Chubbs et al.,^17^ also noted that a few of their patients reported an increased desire to use a higher dose of ketamine, though none misused ketamine through their treatment course. They hypothesized that the desire to use a higher dose of ketamine may also be related to a patient’s perception that the intensity of the dissociative effect is related to the improvement of their depression symptoms, and more ketamine may further improve mood.^17^

While the LCQ reports on liking, craving, and desire to use the prescribed drug (esketamine or ketamine), it does not assess any cravings, desire, or increased use of other substances. Concerns regarding the abuse potential of esketamine largely stem from those stated about ketamine.^17–20^ Ketamine is known to activate the opioid system, which may be important in its antidepressant effects. Due to this activity in the opioid system, it has been cautioned that IN esketamine may lead to abuse liability, and ketamine and esketamine have been likened to opiates.^54,55^ Interestingly however, ketamine treatment has shown promise for the treatment of other substance-related problems—including cocaine, heroin, alcohol abuse, and opioid withdrawal.^14–16,18^ It is unclear whether esketamine would have similar action; however, recent preclinical and clinical trials have shown promising evidence for the management of alcohol and cocaine addiction.^56–58^ Furthermore, somewhat contrary to previous findings that drug users “like” esketamine effects, Chiappini et al.,^59^ reported that patients with comorbid TRD and SUD receiving IN esketamine treatment did not report increased cravings for esketamine. This adds support to the notion that esketamine treatment may have low abuse liability even in individuals with SUD, if used in a therapeutic context.^59^

Only one participant in our study endorsed a strong liking for esketamine, and this remained unchanged after the 8^th^ treatment. No other participants developed this strong “liking,” so this may suggest that individuals prone to “liking” esketamine effects can be identified after the initial treatment. While the Chubbs et al.^17^ study did not assess LCQ scores at multiple time points for ketamine treatment, this would be a future area to explore. Currently, ketamine treatments are less regulated, and there is much debate about the safety of home use. If individuals with a strong liking for ketamine effects can be determined after one treatment, then this information could be used in the consideration of whether the patient is appropriate for home ketamine use. Use of the LCQ in larger populations after the first and eighth ketamine treatments will help elucidate whether this early identification may hold true, or if strong drug liking may develop over the course of treatment.^18^ For instance, by tracking esketamine or ketamine liking and craving through the treatment course, patients reporting increasing LCQ scores could be reassessed by their treatment provider so that individuals developing increased drug liking and/or craving can be identified and necessary treatment modifications can be implemented in a timely fashion. These may include treatment discontinuation, restricting home use, providing a limited supply for home use (i.e., single doses), management of addiction symptoms when appropriate, or effective psychoeducation about the effects of the medication.

### Limitations and future research

Despite these promising results, it is important to consider several limitations and interpret these results with caution. First, though the multicenter open-label observational study design is more generalizable to real-world clinical practice, it also introduces some considerations for the interpretability and validity of the results.^60^ It is unclear why some participants of the original study did not complete the LCQ—this may be due to procedural differences among clinicians or perhaps individuals with a propensity toward abuse elected not to complete the questionnaire.

Similarly, individuals may have underreported ratings on the LCQ. Individuals with high drug liking, cravings, or high desire to use more esketamine than prescribed may not be honest about this, but this is a limitation with any patient-reported scale—much data in psychiatric research relies on the honesty of patient reporting. In this case, patients may fear that higher scores on the LCQ could result in treatment modification or discontinuation.

Individuals with an active SUD were excluded from receiving IN esketamine treatment, and an additional analysis of esketamine likeability and cravings risk was not conducted for any patients in remission from their substance abuse (no SUD for longer than 6 months), as compared to those with no substance abuse history. It is possible that individuals with SUD, either active or in remission, may be at higher risk of desiring to misuse or abuse esketamine when it is delivered at therapeutic doses and thus may have reported higher scores in the LCQ.

Though the patients who discontinued their IN esketamine treatments mentioned “challenges attending regular in-person session appointments, lack of symptom improvement, and personal preferences,” this study did not conduct an additional LCQ assessment for these patients. These results could have elucidated whether an increase in esketamine likeability or craving also impacted these patients’ decision to discontinue their treatments. Some patients may fear liking the drug “too much” and discontinue it for fear of developing a SUD. As such, future studies should aim to collect this data from interested patients.

Another limitation is with the LCQ itself. The LCQ was previously developed considering a comprehensive review of the literature which included the Ketamine Side Effect Tool, and the United States Food and Drug Administration recommendations for evaluation of the addictive potential of a drug (i.e., cravings and likeability),^17,26^ and it has been published and previously used in peer-reviewed published research^17^; but it has not been formally validated. This could impact the applicability and generalizability of the results obtained in this study. This limitation also highlights the need for further validation of effective addiction assessment tools and the scarcity of available tools. The LCQ relies on a visual analog scale for ratings, and other models, such as numerical rating or even a Likert scale, may prove more useful to elicit meaningful responses from participants.

Though including patients without LCQ data is out of the scope of this study, the removal of patients from the original sample (six patients did not report LCQ data) may increase the risk of selection bias and thus should be considered when interpreting the results of this study. Finally, though several patients continued their IN esketamine beyond the acute treatment course (4 weeks), this study did not assess long-term changes in esketamine likeability and craving. This limitation could impact the robustness and generalizability of the findings. Therefore, future studies could consider additional metrics when determining TRD status, and long-term follow-up of patients through their maintenance IN esketamine treatments to assess LCQ scores over time, along with monitoring patients’ substance use patterns.

## Conclusion

The results of this study are promising and support the available literature, showing that baseline levels of drug liking or craving are low for IN esketamine. Further, repeated administration of an acute course of IN esketamine treatment did not increase ratings for likeability or cravings, suggesting that the use of this medication carries low abuse liability. Considering the prevalence of MDD-TRD, and the challenges of managing this condition, these are important results for this patient population and for clinical practice, particularly because most patients require maintenance treatment beyond an index course. While overall results are reassuring, individual patients may remain at risk for substance misuse. The use of a scale such as the LCQ may be useful in clinical practice to assess early identification of drug liking or craving that may precede ketamine or esketamine misuse. While this data does not translate directly to problematic use, it can guide clinicians to follow-up with further clinical assessment. Early detection of proclivity toward esketamine or ketamine misuse can support responsible prescribing, and patient reassessment can support appropriate treatment modifications when needed. Further research can help define risk by repeating the assessment with long-term use, and by distinguishing the motivation behind any desire to use more than prescribed. Future real-world data on drug efficacy and tolerability should include active measures of abuse liability, rather than relying on passive subjective reporting to further support the safety of both ketamine and esketamine in the acute and maintenance treatment courses.

## Supplemental Material

sj-docx-1-taw-10.1177_20420986251347360 – Supplemental material for Abuse liability for esketamine in a cohort of patients undergoing an acute treatment course to manage treatment-resistant depression: a secondary analysis of an observational study in real-world clinical practicee Supplemental material, sj-docx-1-taw-10.1177_20420986251347360 for Abuse liability for esketamine in a cohort of patients undergoing an acute treatment course to manage treatment-resistant depression: a secondary analysis of an observational study in real-world clinical practicee by Gilmar Gutierrez, Gustavo Vazquez, Nisha Ravindran, Raymond W. Lam, Peter Giacobbe, Karthikeyan Ganapathy, Annette Kowara, André Do, Anusha Baskaran, Sean Michael Nestor and Jennifer Swainson in Therapeutic Advances in Drug Safety

## Declarations

**Ethics approval and consent to participate:** The Health Sciences Research Ethics Board at Queen’s University (TRAQ #: 6031788) and institutional review boards at the collaborating sites, reviewed and approved this research study. This study adhered to the standards set by the Declaration of Helsinki, and data management complies with US Federal Health Insurance Portability and Accountability Act (HIPAA) regulations pertaining to patient record confidentiality. Participants in this study provided voluntary, written informed consent for the collection, analysis, and presentation of aggregate data.

**Consent for publication:** Not applicable, only anonymized and aggregate data included in this manuscript.

**Competing interests:** J.S. has received honoraria for speaking for advising/consulting from AbbVie, Bausch Health, Biron, Eisai, Idorsia, Janssen, Lundbeck, Otsuka, and the Canadian Psychiatric Association. R.W.L. has received honoraria for ad hoc speaking or advising/consulting, or received research funds, from: AbbVie, Asia-Pacific Economic Cooperation, Bausch, BC Leading Edge Foundation, Brain Canada, Canadian Institutes of Health Research, Canadian Network for Mood and Anxiety Treatments, Carnot, CB Solutions, Genome BC, Grand Challenges Canada, Healthy Minds Canada, Janssen, Lundbeck, Michael Smith Foundation for Health Research, MITACS, Neurotorium, Ontario Brain Institute, Otsuka, Shanghai Mental Health Centre, Unity Health, Vancouver Coastal Health Research Institute, and VGH-UBCH Foundation. A.D. has received an unrestricted fellowship grant from Janssen Canada and honoraria for ad hoc speaking or advising/consulting, or received research funds, from AbbVie, Canadian Institutes of Health Research, and Otsuka. The rest of the authors declare no other conflicts of interest.

**Availability of data and materials:** All data generated or analyzed during this study is available as needed upon reasonable request.
